# Validity and reliability of a novel monitoring sensor for the quantification of the hitting load in tennis

**DOI:** 10.1371/journal.pone.0255339

**Published:** 2021-07-29

**Authors:** Vedran Hadžić, Aleš Germič, Aleš Filipčič

**Affiliations:** Faculty of sport, University of Ljubljana, Ljubljana, Slovenia; University of Belgrade, SERBIA

## Abstract

Wearable sensor systems are a emerging tools for the evaluation of the sport’s activity and can be used to quantify the external workload of the athlete. The main goal of this paper was to evaluate the validity and reliability of the “Armbeep inertial measurement unit” (IMU) sensor both in a closed tennis exercise and in open matchplay. Twentyfour junior tennis players performed a baseline drill and played matches, during which they wore a combined accelerometer and gyroscope sensor. Video footage was concomitantly recorded using a digital video camera. The agreement between the measurements was assessed with the intraclass correlation coefficient (ICC) and the standard error of measurement (SEM). A simple linear regression was used to predict the number of shots registered from the video and from the Armbeep IMU sensor’s data. The number of total forehand and backhand shots during the drill repetitions showed an excellent test and re-test reproducibility (ICC≥0.90). There was a significant relationship between the Armbeep IMU sensor’s number of contacts and the total number of shots (R^2^ = 0.938) which indicated the excellent reliability of the tested Armbeep IMU sensor for those parameters. Considering the accuracy of the total tennis shots and the small magnitude of error for wrist speed and acceleration, the Armbeep IMU sensor appears to be an appropriate on-court tool that can be used to monitor the hitting load during tennis practice and matches.

## Introduction

A large number of wearable devices, which measure biomechanical, physiological, and other indicators, or recognize sport specific movements of athletes are available on the market. Wearable sports devices incorporating sensor technology most commonly include accelerometers, magnetometers, and gyroscopes, that detect and analyze the sport motor-tasks performed by athletes using inertial measurement units (IMU). Some devices also monitor motor capacity assessment, activity classification and physical demands assessment (heart rate, heart rate variability, and blood oxygen content) [1].

Wearable sensors are increasingly used to measure external load, which is defined as the amount of work performed by an athlete that is measured independently of his or her internal characteristics [2]. In tennis, the external load is represented by running speed and distance traveled as well as shot number, speed, and tempo in a given time unit [3]. Internal load is the athlete’s response to the external load [4]. The assessment of internal training load requires quantification of the intensity and duration of the physiological stress imposed on the athlete [5]. Several methods are used to determine internal training load: heart rate (HR), maximal oxygen consumption (VO_2max_), training impulse (TRIMP), lactate concentrations, as well as biochemical, hormonal, or immunological assessments [6].

As in other sports, there are many different types of performance devices in tennis. These devices can be divided into three groups [7]. The first group includes sensors which are integrated into part of the tennis racket. The second group comprises sensors which the tennis player can attach to the racket handle, and the third is a sensor worn or fastened onto the wrist of the tennis player’s playing arm [7]. Sensors are accompanied by a number of indicators, such as number, type, and speed of shots played [8–10], serve motion analysis [11,12], ball impact location, and post-impact peak force transmitted to the hand [9,13,14], subsequent frame vibrations [13–15], and various types of shots [16]. In addition, sensors can also measure skin temperature and heart rate.

Finding a wearable sensor that would able to monitor both internal and external load is of interest for sports professionals. Tennis currently relies on session count, duration, and stroke analysis to objectively measure external tennis load [17]. In most of the cases, tennis coaches must rely on their intuition, as there is little information for duration, density, or volume of practice [18].The best means for understanding the external load is to monitor and analyse indicators of hitting and movement loads during matchplay or practice [19]. By analyzing the training load of tennis players during various drills, strong relationships have been established between the physical and mental perceptions of training load [17]. Myers at al. [20] found that hitting volume accuracy ranged from 91% to 96% for all forehand, backhand, and overhead shots. Meanwhile, Whiteside at al. [10] concluded that they classified the basic shots (overhead, forehand and backhand) with 97.4% accuracy and the special shots (volleys, shots with rotations) with 93.2% accuracy.

In tennis, the advantages of using IMU sensors could be demonstrated by how they are able to determine possible association between shot volume and risk of overuse injuries, or more accurately, between repetitive tennis shots and the occurrence of upper limb overuse pathologies [21]. Some studies in other sports have examined the relationship between workload and injury in order to identify a workload threshold at which the risk of injury increases [4]. Dannis at al. [22] found that there is an optimal number of throws in a cricket practice within a week. The possibility of injury increases with both, higher and lower than optimal number of repetitions (e.g. optimal load). A study in volleyball [23] has assessed the IMU sensor (The Vert, Mayfonk Athletic, Florida, USA). The number of jump and landing impacts during structured practice and matchplay were analyzed. Validation of wearable sensor was performed to quantify jump loads, where the IMU was utilized to test for jump count and height. Kee studied the reliability of the batting velocity in softball using a small wearable sensor (Zepp Lab, USA) [24]. In the field of tennis, Keaney & Reid [9] tested the accuracy of determination of shot type and impact location and compared these results with racket speed data obtained with the VICON system.

In a commentary in the British Journal of Sports Medicine, Pluim et al. [25] emphasized the need to monitor workload in tennis players as it negatively affects the health of athletes and increases the risk of injury. Therefore, the use of valid, reliable and user-friendly technologies is also necessary in tennis. The Armbeep is a novel monitoring sensor for quantification of hitting load approved as player analysis technology by International Tennis Federation [26]. Novelty of this sensor in comparison to other devices is the fact that Armbeep sensor is worn by an athlete and apart from reporting the shot’s parameters it also provides valuable information regarding the heart rate combining these two training load parameters. The primary objective of this study was to determine the reproducibility, reliability and validity of combined accelerometer and gyroscope in IMU sensor (Armbeep; version 1.0, Biometrika, Maribor, Slovenia) for total shot count against visual inspection in a closed tennis exercise and open matchplay situation [27]. Validity was defined as the extent to which the results really measure what they are supposed to measure [28], and in our case validity represented comparison of Armbeep registered contacts with the number of shots registered from actual video. Primary endpoint in reproducibility and validity analysis was therefore the number of contacts registered by Armbeep. We have also analyzed Armbeep’s secondary outcome measures such as wrist speed and wrist acceleration, contact penalty (effect of racket vibrations on player’s arm) and player’s heart rate during the activity. Testing was performed in practice an open matchplay conditions for all parameters.

## Materials and methods

### Experimental design

This was a cross-sectional reproducibility study designed as a test-retest study, with at least one week off between the two trials. During each trial, participants wore a IMU Armbeep sensor. Players performed (1) a comfort zone drill and (2) an open match play. All activities were concomitantly recorded using a digital video camera. Two independent coaches have evaluated the number of all shots from video (standard procedure used as a gold standard) [29]. Data from Armbeep sensor were exported from both testing days and were compared for reproducibility (test-retest), while validity was assessed comparing gold standard data with IMU sensor data.

### Subjects

Study participants included 24 male and female (mean ±SD 14.3 ±2.5 years, 50.8 ±14.8 kg, and 163 ± 15 cm) adolescent right-handed tennis player volunteers, recruited from a local tennis clubs. Sixteen young boys and 8 girls were involved in the training process for more than 6 years, trained from 8 to 12 hours per week and were ranked on the national junior ranking list. All participants and their legal guardians provided written informed assent and consent. The study was approved by Faculty of sport ethics committee (University of Ljubljana, January 31, 2020, 01–2020).

### Procedures

Data collection was performed on an indoor tennis court with 24 participants using their own rackets while participating in the comfort zone drill [30] and open matchplay, on two occasions (first occasion–test; second occasion–re-test) over a period of 9 days. Participants were familiarized with the drill and testing procedures before testing began. A standardized 15-minute warm-up consisting of dynamic movement and flexibility exercises and tennis specific warm-up, was performed before each testing session.

**Fig 1:**
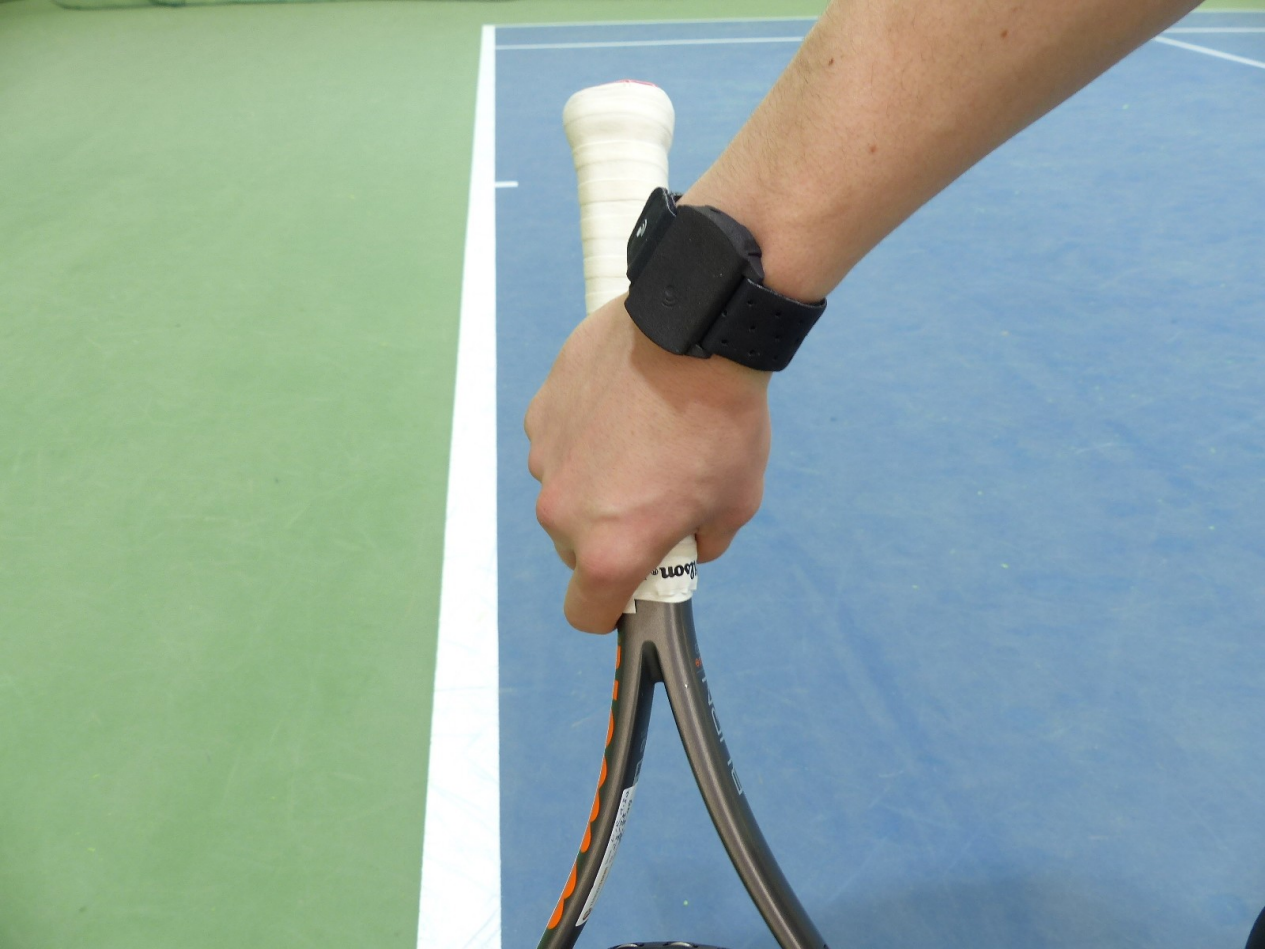
Placement of the IMU.

During training sessions and open game matchplay on hard court surface, participants wore an IMU sensor (mass = 12 g; dimensions = 34 x 29 x 12 mm). All activities were performed with a regular tennis balls (US Open, Wilson Sporting Goods Co., Chicago) and on an indoor tennis court (Rebound Ace GS, California Sport Surfaces, Andover).The sensor was placed laterally, on the ventral surface of the forearm of the hitting arm (Fig 1), and data was collected for the entire duration of the session. IMU sensor collects the accelerometer and gyroscope data in for every shot. Accelerometer readings are measured in all three axes and only movements with amplitude values higher than 1G at the point of impact were recognized as shots [27]. Data collected with the wrist monitoring sensor was transferred to the player’s personal dashboard and exported in CSV format for future data processing.

**Fig 2:**
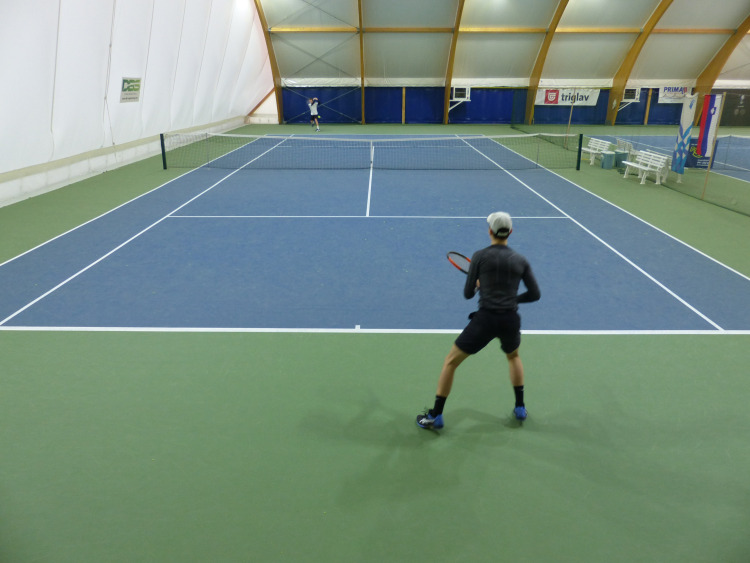
Video camera position during the practice and matchplay.

Video footage of comfort zone drills and open matchplay sessions was recorded using a digital video camera (Panasonic Lumix DMC-FZ100) mounted 2.5 meters high onto a fence behind the baseline, facilitating a view of the entire court (Fig 2). The camera was set to record at AVCHD (13 Mbps) and a resolution of 1280 × 720 pixels. The number of shots played was counted during the comfort zone drill and open matchplay.

The comfort zone drill was chosen as it is known to feature in the training programs of junior tennis players [30]. Groups of two participants completed 12 series of drills which lasted 1 minute. The work and rest intervals lasted 30 seconds and were set in accordance with those commonly used during practice. An experienced professional coach hand-fed new tennis balls to the participant at a frequency determined by the completion of the previous shot and by the movement of the participants to the next shot. All players were instructed by the coach prior to drill to move and hit with maximum effort, aiming all shots at the target areas along the line.

After completing a comfort zone drill on a second occasion the players performed an hour-long open game matchplay. Pairs were selected by coach according to the position on a national ranking list to meet criteria of similar playing standards. Matches were played without chair umpires. Players respected time limits between points (25 seconds), for changeovers (90 seconds), or set breaks (120 seconds). All activities were captured on digital camera and later reviewed by two independent coaches for visual shot count.

All activities were recorded with a digital camera and later reviewed by two independent coaches (with highest tennis coach certification) for visual shot counting. Later, we compared IMU sensor data collected in real time with video data.

### Statistical analysis

All data were calculated using the IBM SPSS Software for Windows (version 21, SPSS Inc., Armonk, New York, USA) and MedCalc (MedCalc Software, Seoul, Republic of Korea). Numeric variables are presented as means and standard deviations. All numeric variables were firstly checked for normality of distribution with Shapiro-Wilk`s test. The differences between test and retest were assessed with repeated measure analysis of variance (ANOVA).

The agreement between measurements (test and re-test) was assessed with the intraclass correlation coefficient (ICC_2,1_), and with the 95% confidence interval (95% CI) for ICC. Values of the ICC are interpreted according to recent guidelines [31]. Absolute and relative measurement error was assessed with standard error of measurement ($\mathrm{SEM}=\mathrm{SD}\times \sqrt{1-ICC}$) and with the SEM %, respectively [32,33].

The qualitative assessment of systematic changes between test and retest means was performed using Bland-Altman plots. These graphs can illustrate the possible issue of heteroscedasticity, which occurs when the test-rest difference increases as the mean of the value of both test decreases [34]. Additionally, the quantitative assessment of heteroscedasticity was calculated with the Pearson`s correlation coefficient or Spearman`s rank correlation coefficient for normally or asymmetrically distributed variables, respectively [35].

Bland Altman plots between the gold standard method (number of shot from video) and the IMU sensor were plotted to assess validity of the new sensor. Additionaly, simple linear regression was used to predict number of shots from video using IMU sensor data. The significance level for all analysis was set at p-values <0.05.

## Results

Our results (Table 1) have shown excellent test-retest reproducibility (ICC≥0.90) for elementary measurement data such as number of shots, forehand and backhand speed and acceleration. Test-retest differences were small and smallest real difference acceptable (<10.5%), indicating the potential for the practical use of IMU sensor as a novel tool for upper arm load monitoring in tennis players. It must be stressed that a derived parameters forehand and backhand contact penalty did not show acceptable reproducibility as 95% confidence interval for ICC was between -0.124 and 0.836 indicating poor to moderate reproducibility, which cannot be acceptable in practice. Contact penalty determines the mechanical effect of the racket vibrations on the tennis player’s arm [36].

**Table 1:** Reproducibility parameters for measurements with Armbeep sensor.

Variables	ICC	95% CI for ICC	d	95% CI for d	CVSD	SEM	SEM %	SRD	SRD (%)
Armbeep total number of shots	0.976	(0.945, 0.989)	-0.05	(0.06,-0.16)	0.042	0.14	0.10%	0.39	0.27
Speed Forehand (m/s)	0.970	(0.933, 0.987)	-0.23	(0.63, -1.09)	2.890	1.19	2.97%	3.31	8.23
Acceleration Forehand (m^2^/s)	0.949	(0.886, 0.978)	-0.10	(0.42, -0.62)	0.999	0.68	3.63%	1.89	10.07
Contact Penalty Forehand (no unit)	0.300	(-0.124, 0.626)	0.02	(0.17,-0.14)	0.094	0.17	12.74%	0.48	35.3
Speed Backhand (m/s)	0.944	(0.876, 0.975)	-0.47	(0.35, -1.30)	2.873	1.19	3.15%	3.29	8.74
Acceleration Backhand (m^2^/s)	0.891	(0.766, 0.951)	-0.30	(0.20, -0.79)	0.881	0.66	3.77%	1.82	10.44
Contact Penalty Backhand (no unit)	0.659	(0.359, 0.836)	0.01	(0.16, -0.14)	0.116	0.22	14.74%	0.61	40.87
Heart Rate (beats/min)	0.645	(0.330, 0.829)	0.35	(6.07, -5.38)	119.49	6.91	4.22%	19.14	11.69

ICC- intraclass correlation coefficient; CI–confidence interval; d–difference between two measurements; CVSD–coefficient of variation of standard deviation; SEM–standard error of measurement; SRD–smallest real difference; SEM/SRD %—the percentage of the mean value.

Upon the retest all measurements were slightly higher, but repeated measures ANOVA did not show any significant differences between test and re-test (p>0.05 for all instances; Table 2). These data suggest that the learning effect is not likely. This was expected as this is a wearable monitor, which should not be influenced by learning effects.

**Table 2:** Repeated measures ANOVA for test and re-test measurements with Armbeep sensor.

	Mean (SD)		Repeated measures ANOVA	
Variables	Test	Re-test	Wilks lambda	p
Armbeep total number of shots	144.16 (0.82)	144.21 (1.02)	0.958	0.328
Speed Forehand (m/s)	40.03 (7.07)	40.40 (6.83)	0.952	0.295
Acceleration Forehand (m^2^/s)	18.77 (3.19)	18.81 (2.93)	0.998	0.827
Contact Penalty Forehand (no unit)	1.36 (0.27)	1.36 (0.24)	1	0.958
Speed Backhand (m/s)	37.46 (5.24)	37.86 (4.93)	0.944	0.256
Acceleration Backhand (m^2^/s)	17.27 (2.03)	17.54 (2.05)	0.923	0.18
Contact Penalty Backhand (no unit)	1.51 (0.47)	1.45 (0.34)	0.969	0.403
Heart rate (beats/min)	164.20 (12.82)	163.33 (12.81)	0.993	0.701

Furthermore, for the good reproducibility of the novel monitoring sensor it is necessary to evaluate indicators of absolute reliability such as SEM, SEM % and SRD. Firstly, load monitoring sensor must have a small measurement error to detect a real change [37]. Our SEM % values were lowest for the number of shots (0.14%), but also, we have obtained SEM % values below 4.5% for speed and acceleration parameters. Once again, a derived parameter contact penalty for forehand and backhand has shown SEM % values of 13% and 15%, respectively indicating a reproducibility issue with those parameters, that were even more stressed with SRD%. That was 35% and 41% for forehand and backhand contact penalty, respectively. In practice, these would mean that a single subject should have increase (or decrease) of that parameter in such range to be detected by such monitor. This is of course once again unacceptable, and we may conclude that measurements of speed, acceleration and number of shots should be enough to quantify upper arm loading with excellent reproducibility and reliability.

Finally, quantitative assessment of the systematic change (Table 3) showed no significant correlation between test-retest means and test-retest difference for number of shots, speed, acceleration, heart rate and forehand contact penalty (r < .30 and p >.05 for all instances). All correlation values were below ±0.263, with the exception of backhand contact penalty (r = 0.436, p = 0.036). Furthermore, the qualitative results via Bland-Altman plots showed good agreement between measurements and homoscedasticity for speed and acceleration (S1 File).

**Table 3:** Correlations between difference and mean of two measurements.

Variables	r	Sig.
Speed Forehand (m/s)	0,14	.515
Acceleration Forehand (m^2^/s)	-0,263	.215
Contact Penalty Forehand (no unit)	-0,098	.650
Speed Backhand (m/s)	-0,187	.383
Acceleration Backhand (m^2^/s)	0,021	.921
Contact Penalty Backhand (no unit)	-0,436	.033
Heart rate (beats/min)	-0,002	.994

**Fig 3:**
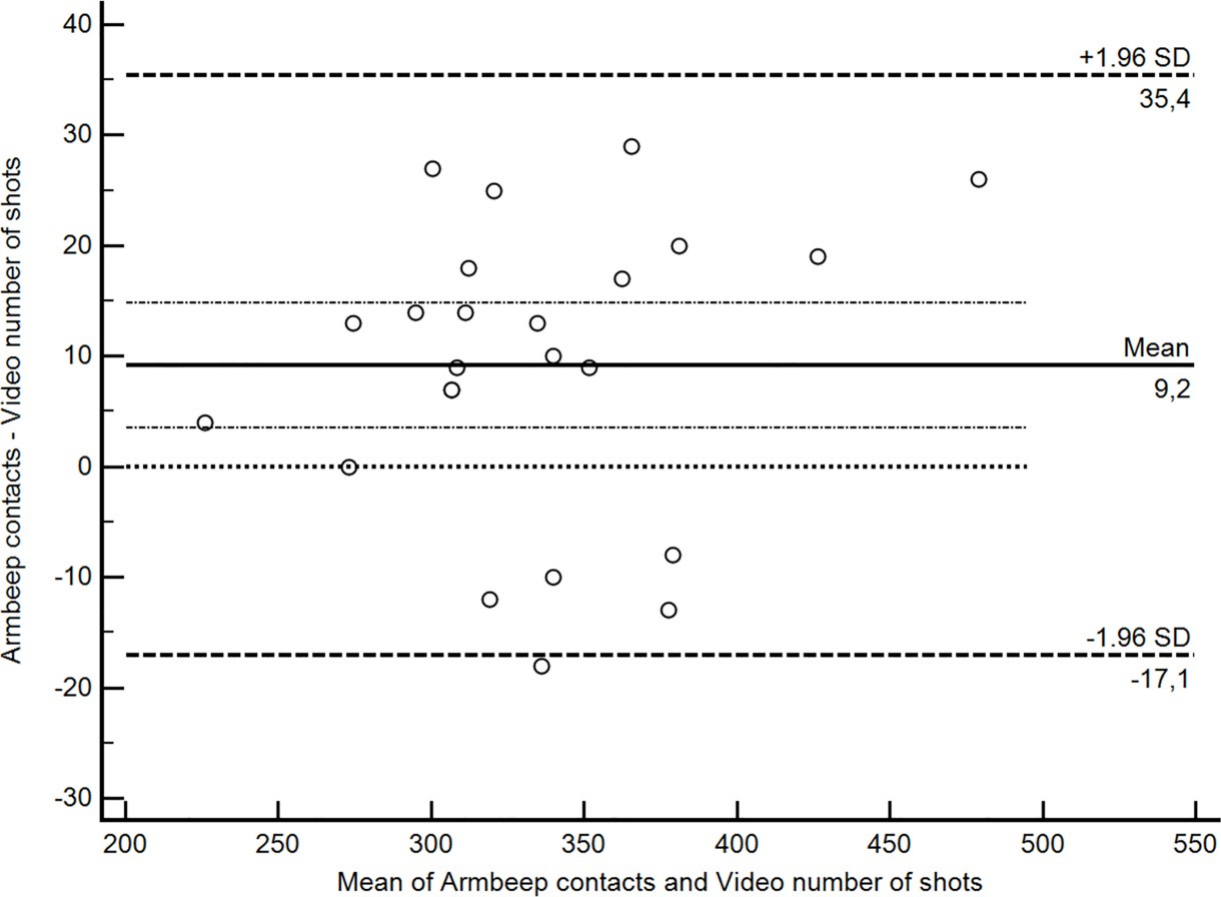
Bland Altman plot of the relationship between mean and difference between Armbeep sensor and video analysis.

The results of match analysis are presented in the Table 4. Fig 3 depicts Bland Altman analysis that was used to validate the Armbeep sensor against the gold standard method. It is clear from the plot that Armbeep sensor tends to overestimate the number of contacts by 9 contacts (95% CI 4–15; p = 0.003). It should be also noted that in 5/25 (21%) cases the Armbeep reported lower number of contacts than was registered from the video (number of circles below zero line).

**Table 4:** Number of contacts from Armbeep sensor and number of shots collected with video analysis during matchplay.

Variables	Mean	Std. Dev.
Armbeep number of contacts	339	54
Total number of shots from video	330	52
Number of non-shots	114	48
Number of shots	216	37
	**Mean**	**% of shots**
First serve (no.)	47	22%
Second serve (no.)	22	10%
Return on second serve (no.)	25	12%
Return on first serve (no.)	15	7%
Forehand (no.)	54	25%
Backhand (no.)	31	15%
Volley Forehand (no.)	2	1%
Volley Backhand (no.)	2	1%
Smash (no.)	2	1%
Slice Backhand (no.)	7	3%
Slice Forehand (no.)	2	1%
Other shots forehand (no.)	4	2%
Other shots backhand (no.)	2	1%

**Fig 4:**
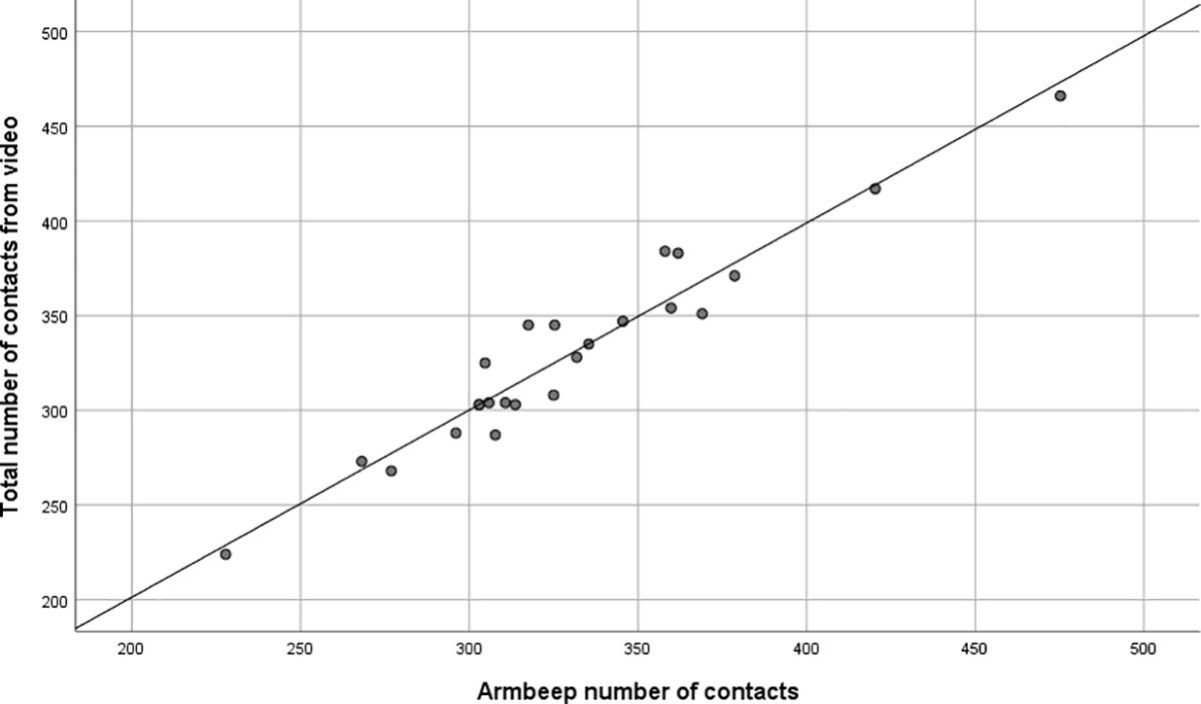
Scatter plot of the relationship between total number of shots collected with video analysis and Armbeep sensor number of contacts.

Simple linear regression was carried out to investigate the relationship between number of shots from video analysis (gold standard) and IMU sensor registered number of contacts. All linear regression assumptions were checked and confirmed. The scatter plot (Fig 4) showed that there was a strong positive linear relationship between the two data collecting methods, which was confirmed with a Pearson’s correlation coefficient of 0.969. Simple linear regression showed a significant relationship between IMU sensor number of contacts and total number of shots (p < 0.001). The slope coefficient for IMU sensor number of contacts was 0.927 (β = 0.969) so the total number of contacts increases by 0.927 contacts for each extra contact registered by IMU sensor (which is completely in line with Bland Altman analysis above). The R^2^ value was 0.938 so 93.8% of the variation in total number of contacts can be explained by the model containing only IMU sensor number of contacts.

## Discussion

This is the first study to assess the validity and reliability of a novel IMU sensor in tennis, which combines two protocols: comfort zone drill and open tennis matchplay. We analyzed and compared the number of contacts collected with IMU sensor in two repetitions of comfort zone drill to asses reliability. For validity analysis we have compared the number of contacts collected with IMU sensor and number of shots collected from video camera during the matchplay.

The comparison of the number of forehand and backhand shots in both comfort zone repetitions has shown excellent test and re-test reproducibility. Values of re-test measurements were slightly higher, but repeated measures ANOVA did not show any significant differences between test and re-test. During the comfort zone test, tennis players executed shots with a constant individual speed and high frequency (12 shots per minute). In this measurement protocol tennis players didn’t have the possibility to execute other types of shots, which could have been detected by IMU sensor, therefore the high reliability is expected.

Variability and dynamic of tennis shots execution distinctly increases during matchplay [38]. In addition to true shots, tennis players during matchplay perform numerous movements, such as stopping, picking up or tapping the ball. The accelerometer in IMU sensor is configured to recognize only shots with amplitude values higher than 1G at the shot’s impact point, which should enable only the detection of true shots. During matchplay tennis players perform certain shots intuitively (return of serves, base line shots after opponents’ error etc.), which represents one third of all contacts detected by IMU sensor. Consequently, in Bland Altman analysis (Fig 3) we can notice overestimation by 9 contacts between Armbeep and video analysis, and such difference is probably caused by such intuitive movements that Armbeep sensor registers, but are not counted during video analysis. Results of linear regression showed significant relationship between the total number of shots recognized by IMU sensor and video analysis. Based on those findings we can conclude that IMU sensor can excellently predict actual number of shots during the tennis match. However, if we assume that for potential shoulder pathology only real shots with enough contacts velocity would contribute to the overall loading than IMU sensor predicts only 18% of such shots (R2 = 0.179, F(1,22) = 4.783, p = 0.04). Meaning that with current settings IMU sensor can reliably record contacts but cannot discriminate well between shots (true) and non-shots (false). However, as true shots represent the 65% of all contacts, and taking in account the fact that total number of contacts that Armbeep registers is a sum of shots and non-shots, we believe that total conctacts could still be a good measure of shoulder loading that could contribute to the development of potential shoulder pathology. Furthermore, the above mentioned mean difference of 9 contacts should be put in perspective of total number of shots during the match, where we can see that 9 contacts are actually less than 3% of all contacts, meaning that such difference would not necessarily interfere with clinical applicability of Armbeep sensor. However, how such margin error would influence quantification of hitting load over a longer period of time (e.g. after many training sessions) should be addressed in future studies.

Considering the total shots number accuracy and small magnitude of error for wrist speed and acceleration, the IMU sensor appears to be an acceptable on-court based tool that can be used to monitor workload during tennis practices and matches. One of the possible uses of IMU sensor is to asses different training protocols which differ from the point of training volume, intensity and content. In addition, the IMU sensor can be used as a training and testing tool. Coaches can use wrist speed and acceleration data on daily basis in order to monitor the progress, as well as to analyze the differences between individual shots (serve, forehand, backhand). In this way, coaches can also compare the results gathered using the IMU sensor with their own observations of kinematic efficiency in players’ technique.

In the study, we wanted to check the possibility of accurately measuring the vibrations that occur at the contact of the racket and the ball and then transmit on to tennis player’s arm. Stuelcken at al. [39] found that lesser skilled players make contact with the ball in off-center locations of the racket more frequently, thereby resulting in racket vibrations of larger magnitudes with each shot. Contact penalty, measured by IMU sensor, determines the quantity of vibrations which occur during the contact between racket and ball. Forehand and backhand contact penalty doesn’t have the appropriate measurement characteristics to accurately determine the vibrations during the shot and thus indirectly allow the assessment of shot quality. It is certainly an important indicator that could contribute to a comprehensive analysis of the characteristics of tennis shots but requires further research. Mainly towards the causes for racket vibrations, with the following possible factors: racket mass, stiffness and velocity, ball contact location, grip stiffness and type of acceleration or deceleration [40].

The IMU sensor as a novel tool could prove to be a useful sensor in the quantification of optimal shots number during practice for genders and specific age categories and performance levels. Providing the measurable and objective data of the specific demands of the tennis may improve understanding of the optimal workload and indirectly change coaches’ training methodology. Also, data collected with Armbeep sensor can provide coaches and researches with the characteristics of players’ long-term development. Data stored in the cloud can be permanently available to the player, coach and parents and represent the player’s training log. For the determination of potential association between number of shots during match and practice prospective studies are needed, if we are to define the cut off points for number of shots at which likelihood for injury rises significantly. We believe that validity and reproducibility findings from our study were necessary for such studies.

One of the study limitations is the likely source of measurement error that exists in the visual review from the video of matchplay. During the match, the player performs movements with a racket, such as false shots (e.g. stopping, picking up the ball or tapping in between points time). It was difficult to determine the boundary between a real shot and a non-shot during the matchplay. Despite the shot recognition problem, the system allows to check timestamp of the shot. In most cases, the IMU sensor recorded more contacts than were recognized during the video analysis. Also, the IMU sensor does not recognize well the type of shot, which is one of the major limitations that could also solve the problem of collecting the total number of shots in matchplay. In addition, the problem of accurate shots recognition is one of the most important areas of automated system for quantifying external hitting load in tennis [10].

Additionally, we have not calculated the sample size for the purposes of this study, but we believe that the sample size used in our study is comparable to previously published study that monitored vertical jump using MyVert sensor in 18 volleyball athletes, who were on average 16.9 years old [41,42]. Apart from that our study included only two observations, that could also be considered as one of the important limitations.

## Conclusions

Based on our results the Armbeep sensor could be considered valid and reliable tool for measuring the actual number of shots during the tennis practice or match under the circumstances described in our study. This is a big step for tennis coaches and players to objectify their workloads during training and competitions, which was so far reduced only to intuitive level. Monitoring the hitting load during all tennis activities is a useful tool for analyzing, monitoring and planning the long-term development of a tennis player. Along with heart rate data, the coaches will also receive data on cardiocirculatory parameters and thus a comprehensive picture of the tennis player’s workload. At the same time, they will be able to compare the workload of a tennis player during matches and training sessions and plan training more accurately based on this information.

## Supporting information

S1 File (DOCX)

S1 Raw data (XLSX)
