# Supplementary material for: Validity and reliability of a novel monitoring sensor for the quantification of the hitting load in tennis
Source: PLoS One. 2021 Jul 29;16(7):e0255339. doi: 10.1371/journal.pone.0255339 (PMC8321100; doi:10.1371/journal.pone.0255339)
Supplement: S1 File — (DOCX) [file pone.0255339.s001.docx]

**Supplementary file 1.** Bland-Altman plots showing the 95% limits of agreement between the test and re-test for different Armbeep parameters

|  |  |
| --- | --- |
|  |  |
|  |  |
|  | |
